# Multiple adaptive routes of *Salmonella enterica* Typhimurium to biocide and antibiotic exposure

**DOI:** 10.1186/s12864-016-2778-z

**Published:** 2016-07-13

**Authors:** Tânia Curiao, Emmanuela Marchi, Denis Grandgirard, Ricardo León-Sampedro, Carlo Viti, Stephen L. Leib, Fernando Baquero, Marco R. Oggioni, José Luis Martinez, Teresa M. Coque

**Affiliations:** Servicio de Microbiología, Instituto Ramón y Cajal de Investigación Sanitaria (IRYCIS), Madrid, Spain; CIBER Epidemiología y Salud Pública (CIBERESP), Madrid, Spain; Unidad de Resistencia a Antibióticos y Virulencia bacteriana asociada al Consejo Superior de Investigaciones Científicas (CSIC), Madrid, Spain; Department of Agrifood Production and Environmental Sciences, University of Florence, Firenze, Italy; Neuroinfection Laboratory, Institute for Infectious Diseases, Bern, Switzerland; University of Leicester, Leicester, UK; Departamento de Biotecnología Microbiana, Centro Nacional de Biotecnología (CSIC), Darwin 3, Cantoblanco, Madrid 28049 Spain

**Keywords:** Biocide resistance, Antimicrobial susceptibility, WGS, Transcriptomics, Collateral sensitivity

## Abstract

**Background:**

Biocides and antibiotics are used to eradicate or prevent the growth of microbial species on surfaces (occasionally on catheters), or infected sites, either in combination or sequentially, raising concerns about the development of co-resistance to both antimicrobial types. The effect of such compounds on *Salmonella enterica*, a major food-borne and zoonotic pathogen, has been analysed in different studies, but only few works evaluated its biological cost, and the overall effects at the genomic and transcriptomic levels associated with diverse phenotypes resulting from biocide exposure, which was the aim of this work.

**Results:**

Exposure to triclosan, clorhexidine, benzalkonium, (but not to hypochlorite) resulted in mutants with different phenotypes to a wide range of antimicrobials even unrelated to the selective agent. Most biocide-resistant mutants showed increased susceptibility to compounds acting on the cell wall (β-lactams) or the cell membranes (poly-L-lysine, polymyxin B, colistin or toxic anions). Mutations (SNPs) were found in three intergenic regions and nine genes, which have a role in energy production, amino acids, carbohydrates or lipids metabolism, some of them involved in membrane transport and pathogenicity. Comparative transcriptomics of biocide-resistant mutants showed over-expression of genes encoding efflux pumps (*sugE*), ribosomal and transcription-related proteins, cold-shock response (*cpeE*) and enzymes of microaerobic metabolism including those of the phosphotransferase system. Mainly ribosomal, metabolic and pathogenicity-related genes had affected expression in both *in vitro*-selected biocide mutants and field *Salmonella* isolates with reduced biocide susceptibility.

**Conclusions:**

Multiple pathways can be involved in the adaptation of *Salmonella* to biocides, mainly related with global stress, or involving metabolic and membrane alterations, and eventually causing “collateral sensitivity” to other antimicrobials. These changes might impact the bacterial-environment interaction, imposing significant bacterial fitness costs which may reduce the chances of fixation and spread of biocide resistant mutants.

**Electronic supplementary material:**

The online version of this article (doi:10.1186/s12864-016-2778-z) contains supplementary material, which is available to authorized users.

## Background

*Salmonella enterica* is a major food-borne pathogen able to cause diarrhoea or thyphoid/paratyphoid fever [1]. The systemic infection is often preceded by an asymptomatic chronic colonization or by a local infection process. One of the major problems associated with persistent colonization or infection is the steady rise of antibiotic resistance among strains, which can lead to treatment failures [2]. The association between the overuse of antibiotics and/or biocides in farms, hospitals, industry and homes and the emergence of both co-resistance and cross-resistance to different compounds in *Salmonella* populations is of concern [3–6].

Unlike antibiotics, most biocides do not act on specific cell targets. In fact, only a few mechanisms by which microorganisms became tolerant to these antimicrobials have been fully characterized. Over-expression of multidrug efflux pumps such as AcrAB or AcrEF which are controlled by global transcriptional regulators such as MarAB, RamA and SoxRS can lead to diverse levels of resistance to biocides and/or antibiotics [7–13]. Often, tolerance to triclosan is due to over-expression and/or mutations in FabI, the enoyl-acyl-reductase protein required for fatty acid synthesis [14]. Moreover, exposure and further adaptation to biocides may also impair cellular homeostasis, and/or changes the level of expression of genes regulating synthesis and modification of cell envelope, virulence, motility, or stress response [15–20]. Whether or not such physiological changes are needed for adaptation to the presence of biocides, or they just reflect secondary changes associated with restoring fitness after adaptation remains to be established. Previous studies in *Salmonella enterica* prototype strain SL1344 have described the modification of antibiotic susceptibility, growth and regulation of different genes after exposure to biocides [5, 6, 21]. However, few studies provided comprehensive information about the genomic and transcriptomic changes of mutants selected after exposure to different biocides and antibiotics, which can be used either coincidentally or sequentially in the clinical practice and in the food industry [9, 22–24].

The aim of this study was to determine the effect of exposure to some biocides (triclosan, TRI; benzalkonium chloride, BKC; chlorhexidine, CHX and sodium hypochlorite, SHC), or antibiotics (ampicillin, AMP; ciprofloxacin, CIP), widely used in farms, hospitals, industry and homes on the selection of antibiotic/biocide-resistant *Salmonella* mutants and to characterize the associated genomic and transcriptomic profiles, as well as the extended phenotypes (susceptibility to 240 inhibitory compounds). To address whether these adaptive changes found in laboratory-selected mutants also occurred in natural populations of *Salmonella*, the transcriptomes of a set of field isolates exhibiting reduced susceptibility to biocides were comparatively studied.

## Methods

### Bacterial strains

The prototype *S. enterica* serovar Typhimurium SL1344 [25] strain was exposed to biocides (TRI, CHX, BKC and SHC), and antibiotics (the β-lactam ampicillin, AMP; and the fluoroquinolone ciprofloxacin, CIP). The quantitative phenotype of this strain against diverse antimicrobials is summarized in Table 1.

**Table 1:** Susceptibility profiles of *Salmonella* mutants respect to SL1344 parental strain

N.°	Pre-conditioning agent	Biocide Phenotype	Biocide MIC (mg/L)			Antibiotic MIC (mg/L)							Mutant designation	Fitness Cost (%)	Frequency of mutation
			TRI	BKC	CHX	AMP	CAZ	CIP	ERY	GEN	CLO	TET			
	-	*Parental strain* SL1344	*0.06*	*16*	*16*	*1.5*	*0.38*	*0.032*	*32*	*1.0*	*3*	*3*			
1	NE	TRI^R^/BKC^R^/CHX^R^	2	32	32	1.5	0.38	0.047	32-48	1.5	3	2	NE/TRI1	11	>1.25E-07
2	NE		0.12	32	32	1	0.25	0.032	32	1.0	4	4	NE/CHX2	34	2.92E-09
3	BKC		0.12	32	32	2	0.38	0.023	32	1.5	3	4	BKC/AMP	-	2.50E-09
4	CHX		0.12	32	32	1	0.5	0.032	48	1.5	2	2	CHX/BKC3	-	1.67E-09
5	BKC	TRI^R^/BKC^R^	0.12	32	16	2	0.38	0.032	48	0.5	4	4	BKC/BKC3	-	2.50E-09
6	CIP	BKC^R^	0.06	32	16	1.5	0.5	0.032	24	1.5	2-3	1.5	CIP/TRI1	More fit	<1.50E-05
7	TRI		0.06	32	16	2	0.5	0.032	32	1.5	3	2-3	TRI/BKC3	31	1.50E-09
8	NE	BKC^R^/CHX^R^/TRI^HS^	0.03	32	32	2	0.38	0.032	32-48	1	4	4	NE/BKC2	-	1.25E-07
9	NE		0.03	32	32	1.5	0.75	0.047	24	1.5	3	1.5	NE/BKC3	more fit	2.08E-11
10	TRI		0.015	32	32	1.5	0.5	0.032	24	1.5	2	1.5	TRI/AMP	-	3.06E-09
11	BKC		0.015	64	32	2	0.38	0.023	32	1	3	4	BKC/CHX2	-	1.67E-08
12	CIP	TRI^HS^/CHX^HS^	0.03	16	8	2	0.38	0.032	32	2	4	3	CIP/CHX1	-	<2.50E-06
13	BKC	TRI^HS^	0.015	16	16	1	0.38	0.023	64	1.5	3	1.5	BKC/CIP	-	1.50E-09
14	CHX		0.015	16	16	2	0.38	0.023	32-48	0.75	3	4	CHX/AMP	17	4.17E-09

NE: non-exposed, - Not done

In the designation of mutants, numbers 1-3 refer to the concentration of compounds in plates as follows: 1- 32 mg/L, 2- 64 mg/L and 3-128 mg/L

Mutants CIP/TRI1 and CIP/CHX1 classified as more fit than SL1344 exhibited -17 % and -21 %, respectively

WGS was performed in the underlined mutants

Sixteen *Salmonella* spp. isolates from food-borne animals with reduced susceptibility to TRI (3 TRI^R^; MIC 1-2 mg/L), BKC (7 BKC^R^; MIC = 128 mg/L), CHX (1 being CHX^R^/BKC^R^, MIC = 16 mg/L (Additional file 1: Figure S1) used in a previous work [26], were investigated for their transcriptomic profiles. Such isolates, collected in a veterinary surveillance project in Europe, showed 13 different PFGE-types and belonged to *Salmonella enterica* subspecies *enterica* [serovars Anatum (n = 8), Hadar (n = 5), Dublin (n = 2)] and subspecies Typhimurium (n = 1).

Most of these strains were susceptible to antibiotics. A few number of isolates harbored plasmids that contained acquired genes coding for resistance to β-lactams (*bla*_TEM-1_), aminoglycosides (*strA*, *strB*), tetracycline (*tetA*, *tetR*) and quinolone (*qepA*). Plasmids from 6 isolates also carried genes encoding resistance to metals (As, Co).

### Selection of mutants

A colony of *S. enterica* serovar Typhimurium SL1344 grown overnight in Luria Bertani (LB) plates was inoculated into LB-broth and LB supplemented with sub-inhibitory concentrations (1/2 × MIC) of biocides (TRI, CHX, BKC and SHC; Sigma-Aldrich, Inc., St. Louis, MO) or antibiotics (AMP and CIP) and further incubated overnight at 37 °C with shaking at 150 rpm. Subsequently, aliquots of 100 μl were plated onto LB plates containing a single biocide or a single antibiotic compound at concentrations ranging 2.5-33 × MIC and incubated at 30 °C. These primary selective plates were examined for growth during 7 days. A variable number of viable mutants (one per colony morphotype per plate) were tested for growth on secondary selective plates containing other biocides or antibiotics. The stability of mutants was evaluated after serial passages in non-selective LB broth (up to 50 generations). Mutants were named by the acronym name of the antimicrobial compound added to the broth cultures before plating, followed by the name and concentration of the compound added to the selective agar plates from where the mutant was retrieved. The mutants obtained from broth cultures not supplemented with any antimicrobial were designated as non-exposed (NE). Phenotypes of decreased and increased susceptibility to biocides and antibiotics appear represented by the super indexes “R” or “HS”, respectively. The colonial morphology in LB and blood agar plates was compared between mutants and the parental strain. The variability of *Xba*I-digested genomic DNA profiles of mutants and parental strain was assessed by pulsed field gel electrophoresis (PFGE) using standard protocols for DNA preparation, digestion and PFGE running conditions for *Enterobacteriaceae* [27].

### Antimicrobial susceptibility testing

The minimal inhibitory concentrations (MICs) of biocides (TRI, CHX, BKC) and antibiotics (AMP; CAZ; CIP; erythromycin, ERY; gentamicin, GEN; chloramphenicol, CLO; tetracycline, TET) (BioMérieux, Marcy l’Etoile, France) were determined by both broth micro-dilution using E- test strips following CLSI guidelines. *Escherichia coli* ATCC10536 and *Staphylococcus aureus* ATCC6538 were used as control strains (3). Minimum bactericidal concentrations (MBCs) were determined by subculturing 10 μl from each well without visible bacterial growth when MIC was determined on Mueller-Hinton broth (Difco, Becton Dickinson, Maryland, USA). The minimal concentration yielding no-growth after overnight incubation at 37 °C was scored as the MBC. The susceptibility of wild type strains to AMP, streptomycin, sulphonamides, trimethoprim, nalidixic acid, CIP, CLO, TET, GEN and kanamycin was determined by disk diffusion.

Further, the susceptibility of mutants and the parental strain to 240 cell growth-inhibiting chemical compounds was screened using the Phenotype MicroArray PM11-PM20 in two independent experiments (Biolog, Hayward, CA, USA) as previously described [28]. The strains were grown overnight at 30 ° C on BUG agar (Biolog Universal Agar, Biolog Hayward California) and then, colonies were picked up with a sterile cotton swab and suspended in 15 ml of 1X inoculation fluid (IF-0a GN/GP Base, Biolog 74268). Cell density was adjusted to 85 % transmittance (T) on a Biolog turbidimeter. Inoculation fluid for PM11-20 was prepared mixing 100 ml of IF-10a GN Base (1.2X) (Biolog 74264), 1.2 ml of Biolog Redox Dye A (100X) (Biolog 74221), 0.6 ml of cell suspension at 85 % T, bringing to a final volume of 120 ml with sterile water. The mixture was inoculated in the PM plates (100 μl per well) and monitored automatically for color development every 15 min for 72 h at 30 °C in an Omnilog reader (Biolog). To identify phenotypes, the kinetic curves of both parental strain and mutants were compared using Omnilog-PM software (release OM_PM_109M). Such comparison was based on the half maximal inhibitory concentration (IC50) values for 4 concentrations of each antimicrobial, which is defined as the well at which a particular per-well parameter is the half of its maximal value over the concentration series; the reference parameter being the area under the curve. Raw data were filtered using differences of average area of mutant compared to control taking a difference of 1:3 (33 %) as significant.

### Growth kinetics

The growth kinetics of both the parental strain SL1344 and biocide-tolerant mutants exhibiting various phenotypes was determined by measuring the optical density at 600 nm every 5–10 min for 24 h at 37 °C in Bioscreen C (ThermoLabsystems, Helsinki, Finland), adapting the method described by Foucault *et al*. [29]. Inocula in a concentration of 10^4^ to 10^5^ CFU/ml were obtained from a 1/1000 dilution of an overnight culture in fresh LB broth and aliquots of 400 μl were seeded in triplicate in a microtitre plate. Growth rates were determined in the interval estimated to be exponential using the GrowthRates 2.1 program [30]. The fitness cost (FC) reflects the relative growth rates, which were based on the individual growth rates of mutants relative to the parental strain. For each strain, data from growth rates were averaged and standard deviations calculated.

### Whole genome sequencing (WGS)

Six mutants with different phenotypes were selected for whole genome sequencing. Genomic DNA was extracted from 1 ml of overnight cultures in plain LB broth using a Promega Wizard Genomic DNA Purification kit according manufacturer instructions. Genome sequencing was performed on Illumina MiSeq platform to obtain 100–200 bp paired-end reads. Reads were revised and corrected using Lighter software and further mapped against the genome of the SL1344 strain (GenBank acc. Number FQ312003) using Breseq v0.26.1 pipeline (http://barricklab.org/twiki/bin/view/Lab/ToolsBacterialGenomeResequencing). Single nucleotide polymorphisms (SNPs) detected in all mutants were not deemed confident and were excluded, because we cannot dismiss differences between the laboratory SL1344 strain used as wild-type in this work, and that corresponding to the canonical sequence in the GenBank database. Reads not found in all mutants were treated as deletions.

### Transcriptome analysis

#### Array Design and Production

An array was designed to cover the complete genome of *Salmonella enterica* subsp. enterica serovar Typhimurium, as well as plasmids isolated from various Gram-negative microorganisms. Probe design was performed by the CustomArray Design Service (CustomArray Inc., Bothell, WA, USA) and included 12,005 capture probes (35–40 bp length), 326 quality control probes and 65 non-specific probes derived from plants, phages and unrelated bacterial sequences, and also 148 empty spots with no oligonucleotides. Arrays were synthesized on a CustomArray Synthesizer (CombiMatrix, Mukilteo, WA) and quality tested using the standard protocols provided by the manufacturer.

#### RNA extraction

Strains were grown overnight in 10 ml LB broth at 37 °C, 150 rpm. The cultures were diluted 1:100 in pre-warmed LB and grown to logarithmic phase (OD_570_ = 0.5). 2 ml of the culture (5 x 10^8^ – 1 x 10^9^ colony forming units (UFC)/ml) were harvested in 4 ml of RNA protect reagent® (Qiagen GmbH, Hilden, Germany), incubated for 5 min at room temperature and centrifuged for 10 min at 5000 x g. Bacterial pellets were suspended in 200 μl of TE buffer (10 mM Tris/HCl, 1 mM EDTA, pH 8) containing 1 mg/ml lysozyme (Sigma) and incubated for 5 min at room temperature, 600 rpm. Total RNA was then extracted using RNeasy Mini Kit (Qiagen), according to the manufacturer’s instructions. Contaminating DNA was removed using DNA-free™ Kit (Applied Biosystems). The RNA isolation procedure was validated for RNA quality by testing RNA samples on an Agilent 2100 Bioanalyzer (Agilent Technologies). RNA concentration and purity were determined by Nanodrop® ND-1000 spectrophotometer (Thermo Scientific). For each strain, at least 4 RNA samples were prepared from independent cultures.

#### RNA labelling and fragmentation

Isolated, unamplified RNA was labelled with Cy5, using ULS™ Labeling Kit for CombiMatrix arrays (Kreatech Biotechnology), according to the manufacturer’s instructions. RNA was fragmented with the RNA Fragmentation Reagents (Ambion®).

#### Array hybridization

12 K Custom arrays were hybridized with 2 μg of labelled, fragmented RNA, according to information provided by the manufacturer (Customarray/Combimatrix Incorporated). In brief, after pre-hybridization of the arrays, hybridization was performed at 45 °C for 16 hours in a hybridization buffer containing 25 % formamide. After washing steps, microarrays were scanned using Packard ScanArray4000 array scanner and software (ScanArray, version 3.1, Packard BioChip Technologies) with incremental laser power from 15 to 100 %. Data were extracted with Microarray Imager software (version 5.8.0, Combi Matrix) and spot intensity expressed as median intensity. After scanning, microarrays were striped using 12 K CustomArray™ Stripping kit, according to the manufacturer’s instructions. Quality of the stripping was verified by scanning the microarray at maximal laser intensity and repeated when necessary. Microarrays were used up to four times.

#### Data analysis

To adjust for difference in the amount and labelling efficiency of hybridized RNAs, the median fluorescence intensity values of all spots was determined for all laser intensities used during scanning. Scanning data with similar median fluorescence intensity were chosen for further analysis. Fluorescence values of spots with maximal intensity (signal saturation) at a given laser intensity were extrapolated by linear regression, using values gathered with lower laser intensity. For each set of arrays for a given strain, non-specific binding was determined from fluorescence values of the non-specific probes. The cut-off for specific binding was set as the upper 95 % confidence interval of the mean signal intensity of the non-specific probes. Probes were excluded when the mean values for the strains compared were under the cut-off value.

The fluorescence values were log_2_ transformed and stage-wise quantitative normalization was performed for each set of comparison, using a script written in the statistical computing environment of R (R Development Core Team, 2011, version 3.3). To identify genes differentially regulated, we analyzed the transformed and normalized intensities determined by two methods, the Significance Analysis of Microarrays method (SAM, version 5.0, running under Shiny, a web-based interactive application framework for R environment, https://github.com/MikeJSeo/SAM) and “R”, comprising base package statistics and the attached LIMMA package (version 3.26.5). The presence of genes identified by both methods in the mutants analyzed was searched in the wild type strains included in the study and transcriptomic profiles were compared. The expression profiles of these genes were visualized in a heatmap built with the ‘pheatmap’ package in “R”.

#### Statistical analysis

In the SAM method, the delta value was set to obtain an average. False Discovery Rate (FDR) of 5 % and the fold change cut-off value was established as 1.5. In LIMMA analysis, genes with a fold change >1.5 and *p* < 0.05 were considered as differentially expressed. Only the genes identified as differentially expressed by both SAM and LIMMA were considered.

#### Availability of supporting data

The data sets supporting the results of this article are available in the ArrayExpress repository, (http://www.ebi.ac.uk/arrayexpress/) under accession numbers A-MEXP-2366 (*S.* Typhimurium combimatrix 12 K customarray design) and E-MTAB-2554 (microarray raw results).

## Results

### Exposure to biocides or antibiotics yield mutants with different susceptibility to biocides and antimicrobials

Table 1 shows the diversity of mutants exhibiting phenotypes obtained (4 TRI^R^/BKC^R^/CHX^R^, 1 TRI^R^/BKC^R^, 4 BKC^R^/CHX^R^/TRI^HS^, 2 BKC^R^, 2 TRI^HS^ and 1 TRI^HS^/CHX^HS^). Some mutants with increased MICs to biocides were obtained without previous exposure to any antimicrobial but using selective plates supplemented with TRI, BKC or CHX. Others were retrieved after exposure to antibiotics such as CIP and AMP. Previous exposure to SHC did not yield resistant mutants. A number of biocide tolerant mutants mentioned above showed lower MIC values for AMP, CAZ, CIP, ERY, GEN, CLO and TET than those for the corresponding parental strain (<2-fold).

Preexposure to sub-inhibitory concentrations of BKC resulted in mutants with either decreased susceptibility to BKC, TRI and/or CHX (TRI^R^/BKC^R^/CHX^R^, TRI^R^/BKC^R^) or increased susceptibility to TRI (TRI^HS^, BKC^R^/CHX^R^/TRI^HS^). Two of these four mutants selected in plates supplemented with BKC also showed a slight increase in MIC_CAZ_ (1 BKC^R^ and 1 BKC^R^/CHX^R^/TRI^HS^). The TRI^R^/BKC^R^/CHX^R^ and the TRI^HS^ phenotypes were also selected after pre-exposure to CHX, CIP or without pre-exposure to any antimicrobial. A hyper-susceptible TRI^HS^ mutant (BKC/CIP) showed a 2-fold increased MIC_ERY_.

Preexposure to sub-inhibitory concentrations of TRI resulted in mutants that only showed the phenotypes BKC^R^ and BKC^R^/CHX^R^, which could even exhibit increased susceptibility to TRI. Such BKC^R^ mutants showed a minor increase in MIC values to CAZ. Two different mutants were selected on plates supplemented with TRI, one obtained without previous exposure to antimicrobials showed an increased MIC_TRI_ (33-fold) and small increases in MIC_CIP_, MIC_ERY_ and MIC_GEN_. The other, obtained after pre-conditioning with CIP did not show an increase in MIC_TRI_. Pre-exposure to CIP resulted on mutants showing BKC^R^ or TRI^HS^ phenotypes, which were closely related with the above ones.

A more comprehensive analysis of the effects that the exposure to biocides and antimicrobials had on *Salmonella* strain SL1344 strain was performed by characterizing the genome and transcriptome of six mutants representing the phenotypes TRI^R^/BKC^R^/CHX^R^ (CHX/BKC3, NE/TRI1, NE/CHX2), BKC^R^/CHX^R^/TRI^HS^ (NE/BKC3), BKC^R^ (TRI/BKC3), and TRI^HS^ (CHX/AMP) (Table 2).

**Table 2:** Techniques carried out for a representative subset of mutants

Mutant n°	Mutant name	Biolog (Table 3)	WGS (Table 4)	Gene expression (Fig. 1 and Fig. 2)
1	NE/TRI1	Yes	Yes	Yes
2	NE/CHX2	No	Yes	Yes
4	CHX/BKC3	Yes	Yes	Yes
7	TRI/BKC3	Yes	Yes	Yes
9	NE/BKC3	Yes	Yes	Yes
10	TRI/AMP	Yes	No	No
11	BKC/CHX2	Yes	No	No
14	CHX/AMP	Yes	Yes	Yes

Yes and No denotes whether the technique was performed or not

### Antimicrobial susceptibility

We identified mutants with a given biocide phenotype and variable antibiotic susceptibility patterns but also mutants exhibiting different biocide susceptibility phenotypes and similar antimicrobial susceptibility profiles (Table 3). The activity of different compounds against seven mutants and the parental strain was evaluated considering their IC50 values. High IC_50_ values were observed for antibiotics that inhibit protein synthesis (e.g. neomycin and thiamphenicol), specific metabolic routes as the reduction of dihydrofolic acid to tetrahydrofolic acid, which is an essential precursor in the thymidine synthesis pathway (trimethoprim), or membrane acting compounds (toxic cations, such as antimony (III) chloride). Conversely, compounds that act on the bacterial cell envelopes (such as colistin, β-lactams, poly-L-lysine, polymyxin B), toxic anions (e.g. potassium chromate), quaternary salts (e.g. sodium metaborate) and protease inhibitors showed low IC_50_ values.

**Table 3:** Antimicrobial susceptibility determined by BIOLOG for *Salmonella enterica* mutants in comparison to the parental strain

		TRI^R^/BKC^R^/CHX^R^		BKC^R^	BKC^R^/CHX^R^/TRI^HS^			TRI^HS^
Chemicals	Inhibitor Family	NE/TRI1	CHX/BKC3	TRI/BKC3	NE/BKC3	TRI/AMP	BKC/CHX2	CHX/AMP
ANTIBIOTICS								
Neomycin	aminoglycosides	R	R	R	R	R	R	S
Paromomycin		S	S			S	S	
Sisomicin		S	S	R	R	S	R	R
Chloramphenicol	amphenicols		S	S	S			
Thiamphenicol		R	R		R	R		R
Cefazolin	cephalosporins		R					
Ceftriaxone					S			
Amoxicillin	lactams	S	R	S	S	S	R	R
Aztreonam		S		S				S
Carbenicillin		S		S			S	
Carbenicillin (II)						R		
Phleomycin	DNA oxidants						R	
Cinoxacin	DNA topoisomerases inhibitors	S		S	S	S	S	R
Ciprofloxacin		R		R			R	R
Enoxacin		R	S	S	S	S	S	R
Nalidixic acid		R	S	S	R	S	S	R
Novobiocin		S						
Ofloxacin			S	S		S	S	S
Pipemidic acid		R						
Hydroxyurea	folate antagonists	R	S	R	S	R	R	S
Trimethoprim		R	R		R	R		R
Troleandomycin	macrolides		R	R			R	
Rifampicin	RNA polymerase inhibitors			S				
Penimepicycline	tetracyclines		S	S	S	R	S	
Tetracycline				S				
NON-ANTIBIOTICS								
1-Hydroxypyridine-2-thione	chelators			S	S		S	
5,7-Dichloro-8-OHquinoline			S	S			S	
5-Chloro-7-iodo-8-OHquinoline		S	R	S	R	S		S
8-Hydroxyquinoline			R	R			R	R
Fusaric acid		S		S	S		S	
2-Phenylphenol	DNA intercalators						R	
Chloroxylenol	Fungicides					R		
Patulin			R	R			R	R
Colistin	Membrane active agents	S	S	S	S	S	S	R
Polymyxin B		S	S	S	S			
Polymyxin B (II)							R	
Poly-L-lysine		S	S	S	S	S	S	S
Alexidine		S						S
Ornidazole	oxidizing agents	S			S		S	
1-Chloro-2,4-dinitrobenzene		S	S	R	S		R	R
Atropine	other drugs		S		S			
Pridinol		R	R	R		R	R	
Propranolol			S	S	S	S	S	S
Chlorpromazine			S	S	S	S	S	S
Benzethonium chloride	QACs	S	S	S	R	S	S	R
Cetylpyridinium chloride								S
Sodium azide	respiration, uncoupler			S				
Thioridazine				S	S			S
Potassium chromate	toxic anions	S	S	S	S		S	R
Potassium tellurite (II)		S	R		R	R	S	S
Sodium metaborate		S	S	S	S	S	S	S
Sodium tungstate						R		
Sodium periodate				R			R	
Antimony(III) chloride	toxic cations		R	R	R	R		R
Cadmium chloride		R			R			
Thallium(I) acetate				R				R
D,L-Methionine hydroxamate	other inhibitors	S	S	S	S	S	S	S
Phenyl-methylsulfonyl-fluoride		S		S	S	S	S	S
D-Serine		R						
6-Mercaptopurine			S	S		R		
Compound 48/80					S	S		

Blank cells indicate that no variation in the susceptibility were found in comparison to the parental strain

Within this common antimicrobial susceptibility profile, differences were observed for some mutants. The two TRI^R^/BKC^R^/CHX^R^ (CHX/BKC3, NE/TRI1) and the BKC^R^/CHX^R^/TRI^HS^ (NE/BKC3) exhibited an increased susceptibility to the oxidizing agent 1-chloro-2,4-dinitrobenzene, and the tRNA synthetase inhibitor D,L-methionine hydroxamate. The two mutants exhibiting decreased susceptibility to CIP by standard MIC testing, TRI^R^/BKC^R^/CHX^R^ (NE/TRI1) and BKC^R^/CHX^R^/TRI^HS^ (NE/BKC3) also showed resistance to other quinolones such as nalidixic acid and/or enoxacin. Those with increased susceptibility to TRI [BKC^R^/CHX^R^/TRI^HS^ (NE/BKC3) and TRI^HS^ (CHX/AMP)] were more tolerant to benzethonium chloride than the parental strain. This compound is a synthetic QAC widely used in different settings, including the food industry as a hard-surface disinfectant, antiseptic, and in foaming hand sanitizers [31]. It is noteworthy that resistance to TRI in *Salmonella* was frequently associated with tolerance to several antibiotics of different families, with the exception of aminoglycosides.

### Changes in the genome of SL1344 strain after exposure to biocides

The six SL1344 mutants fully sequenced showed SNPs and deletions in genes involved in cell division/stress, membrane transport, cell motility, metabolism and virulence (Table 4).

**Table 4:** Mutations identified in sequenced mutants

		TRI^R^CHX^R^BKC^R^				BKC^R^	TRI^HS^CHX^R^BKC^R^	TRI^HS^
Function	Gene	NE/TRI1	NE/CHX2	CHX/BKC3		TRI/BKC3	NE/BKC3	CHX/AMP
Amino acid/peptide transport/metabolism	*asnA* ^a^						G → T/A74A	
Energy production/amino acid metabolism	*aarF* ^b^						T → A/L317Q	
Lipid metabolism	*fabI* ^c^	G → A/G93S						
Membrane/transport	*tolA* ^d^							T → A/V168E
Replication	*ftsK* ^e^	T → G/P772P		A → T/Q780H	A → T/Q793H			
Surface structures	*mipA* ^f^						T → C/F61L	
	*motB* ^g^					T → G/E128D		
Virulence	*bigA* ^h^		Deletion:408 bp			Deletion:331 bp		Deletion:293 bp
Intergenic	-/- ^i^							T → A
	*purH* ^j^ */-*		A → C				A → C	
	*tdcA/garA* ^k^					C → T		
Unknown	*yeaN* ^l^			C → G/T321T				

Description of gene products: ^a^ Asparagine synthetase A, ^b^ Ubiquinone biosynthesis protein; ^c^ Enoyl-acyl carrier protein reductase (NADH); ^d^ Membrane transporter TolA protein; ^e^ Cell division protein FtsK; ^f^ Hypothetical outer membrane protein; ^g^ Motility protein B; ^h^ Hypothetical surface-exposed virulence protein; ^i^ Intergenic region between Glutamyl-t-RNA synthetase (459 bp)/(359 bp) Xanthosin operon transcriptional regulator; ^j^ Intergenic region between Phosphoribosylaminoimidezolecarboxamide fosmyltransferase and IMP cyclohydrolase/- (16S rRNA); ^k^ *tdc* operon transcriptional activator/Hypothetical surface-exposed virulence protein; ^l^ Hypothetical membrane protein of Major Facilitator Superfamily (transporter)

The three TRI^R^/BKC^R^/CHX^R^ mutants (NE/TRI1, CHX/BKC3, and NE/CHX2) differed in the number and nucleotide changes. The NE/TRI1 mutant presenting the highest MIC_TRI_ value (2 mg/L) had a SNP in the *fabI* gene that resulted in the Gly93Ser change in the binding site of FabI. It also presented a SNP in the *ftsK* gene, which is involved in cell division and likely in the last steps of peptidoglycan biosynthesis. The CHX/BKC3 mutant harboured two mutations in the *ftsK* gene and a SNP in the *yeaN* gene, which encodes a still uncharacterized transporter of the Major Facilitator Superfamily (MFS). Finally, the NE/CHX2 mutant showed a SNP in the intergenic region in the boundary of *purH* and the 16S rRNA genes, the former related to purine metabolism and previously associated with virulence in different bacterial species.

The BKC^R^/CHX^R^/TRI^HS^ (NE/BKC3) mutant showed unique SNPs in *mipA*, *asnA*, *aarF* genes, but the same SNP described above for NE/CHX2. MipA is an outer membrane protein possibly involved in a novel antibiotic resistance mechanism against kanamycin in *E. coli* [32]. The other genes are related to the metabolism of amino acids and ubiquinones.

The TRI^HS^ mutant (CHX/AMP) showed an amino acid change in TolA, the membrane protein specialized in colicin uptake which has been previously involved in detergent tolerance [33], along with a mutation in the intergenic region between the glutamyl-t-RNA synthetase (459 bp)/(359 bp) and the regulator of the xanthosin operon transcriptional regulator.

The genome of the BKC^R^ mutant (TRI/BKC3) showed three changes located at the nucleotide sequence of *motB* gene, the intergenic region next the *tdc*/*garA* operon and a deletion in the *bigA* gene. The *motB* (and *motA*) gene encodes flagellar motor proteins that generate the stator and proton channel anchored to the peptidoglycan layer. The *tdc* operon is a single transcriptional unit involved in threonine and serine metabolism during anaerobic growth. Finally, deletions in the *bigA* gene encoding a hypothetical surface/exposed protein were associated with virulence in some intracellular pathogens [34]. Deletions in *bigA* gene were also found in other two mutants showing TRI^HS^ (CHX/AMP) and TRI^R^/BKC^R^/CHX^R^ (NE/CHX2) phenotypes.

### Changes in the transcriptome of *S. enterica* SL1344 biocide resistant mutants

**Fig. 1:**
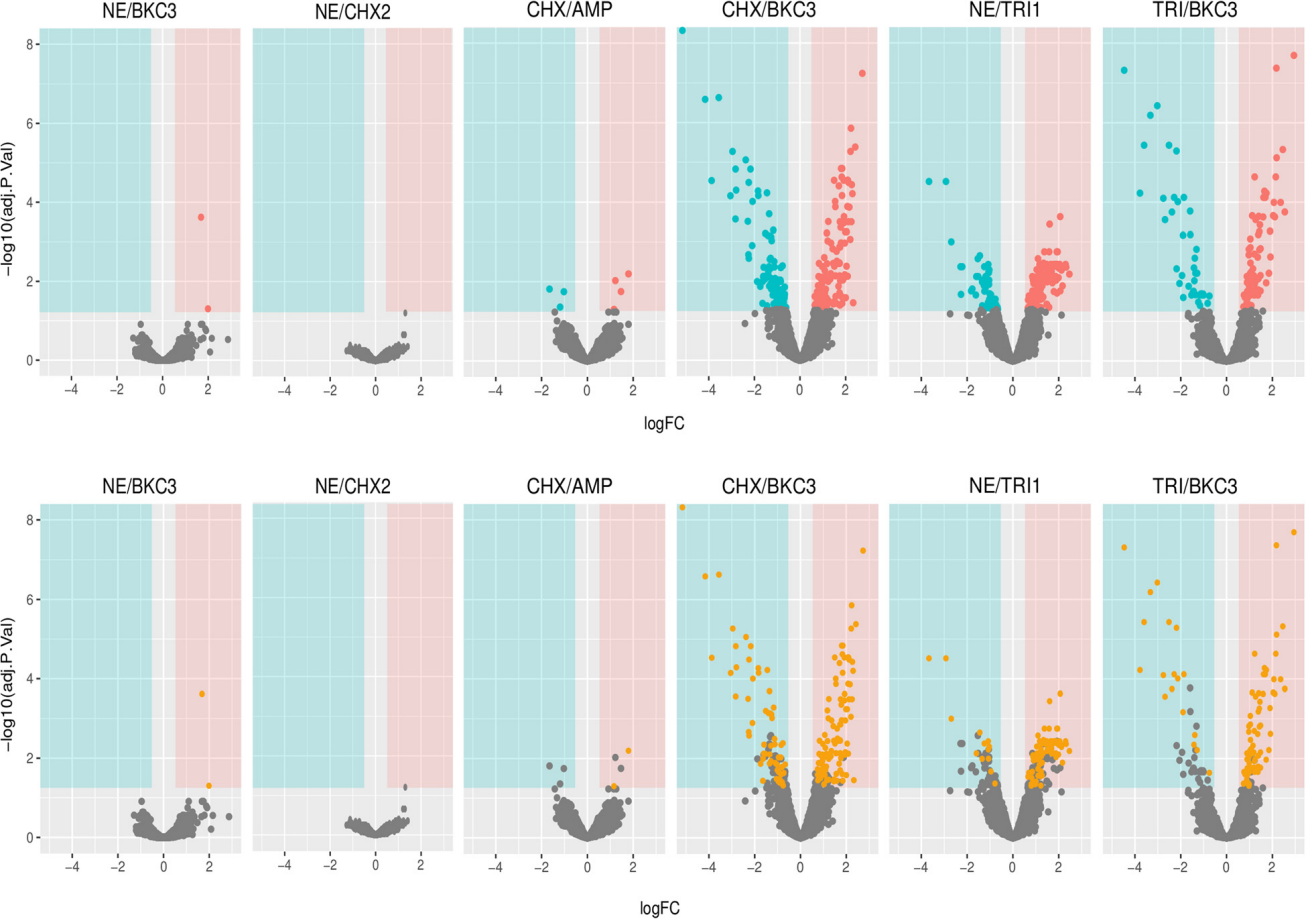
Volcano plots of genes showing altered expression after exposure to biocides in the six mutants analyzed. Log2 fold changes and their corresponding *p*-values of all genes in the microarray were considered to build the Volcano plot. Genes up-regulated (>1.5 fold change, *p*< 0.05) are depicted in *red box*. Genes down regulated (>1.5 fold change, *p* < 0.05) appear in *blue boxes*. Genes that did not show a significantly modified pattern are represented in *grey dots* and the *yellow dots* depict the genes found in both SAM and LIMMA outputs for each mutant

**Fig. 2:**
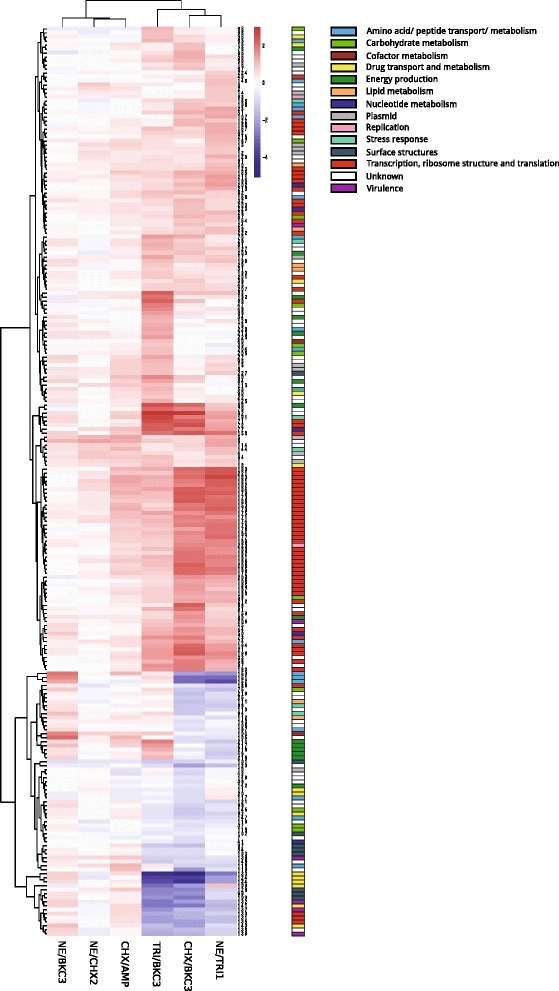
Heat map of the genes with altered expression in different mutants

Figure 1 shows the Volcano Plots of genes altered after exposure to biocides in the six mutants analyzed and Fig. 2, the heatmap clustering mutants according to the expression profiles of genes identified as differently regulated using both methods (LIMMA and SAM). Down regulation of genes was more remarkable than overexpression of genes (-4 fold vs 2 fold Additional file 2: Table S1).

Differential expression was identified for genes involved in cellular metabolism (carbohydrate, lipid, amino acid, and cofactors), energy production, ion transport/metabolism, protein synthesis and signal chemotaxis pathways (Additional file 3: Table 2). The expression of genes classically involved in tolerance to biocides as efflux-pumps (*tolC*, *sugE*) was slightly increased in some mutants.

Two TRI^R^CHX^R^BKC^R^ mutants (CHX/BKC3, NE/TRI1) and one BKC^R^ (TRI/BKC3) showed similar gene expression profiles. Upregulation of genes involved in protein synthesis occurred in these three mutants, and some other changes were common, either in three or two of them, involving genes related to transcription such as *rpoZ* and *rpoB* which encodes subunits of the RNA polymerase. Conversely, transcriptional regulators were repressed in NE/TRI (*malT*) and CHX/BKC3 and TRI/BKC3 (*hilC*, *hilD*, *sprB*). The maltose monomeric activator MalT is a LuxR/UhpA member, a family of large size proteins believed to have another functions (e.g. amino acid metabolism). The others are members of the AraC family and negative regulators of HilA, the key regulator of the *Salmonella* pathogenicity island 1 (SPI1) [35, 36].

Both TRI/BKC3 and CHX/BKC3 mutants shared a pattern of down-regulation of genes related to transport and metabolism with a potential role in virulence such as some within type III secretion system complex, e.g. *prgI*, *pgrH* and *sopE* or possibly *ompC*, encoding surface proteins involved in chemotaxis signal transduction system *i.e.* part of flagella and invasion proteins (*iagB* and *orgA*). The resultant proteins regulate the events that lead to changes in the swimming behaviour of the cell [37]. Moreover, the two mutants NE/TRI1 and CHX/BKC3 distinguished from the rest by the down-expression of genes linked to threonine metabolism (*tdcB* and *tdcC*).

Overexpression of genes coding for metabolic enzymes related to energy production such as glucose catabolism (GapA pyruvate kinase, fumarate reductase), some PTS systems and other carbohydrate metabolic components were shown for TRI/BKC3.

Genes with a function related to some of those with mutations such as *ftsl2* and *ftsH*, *yeaD*, *yeaC* and *yeaF*, *tdcB*, *tdcC* and *tdcE* were found to have altered expression in several mutants.

### Similar transcriptomic profiles were exhibited by field *Salmonella* strains showing variable reduced susceptibility to biocides and the laboratory-selected mutants

**Fig. 3:**
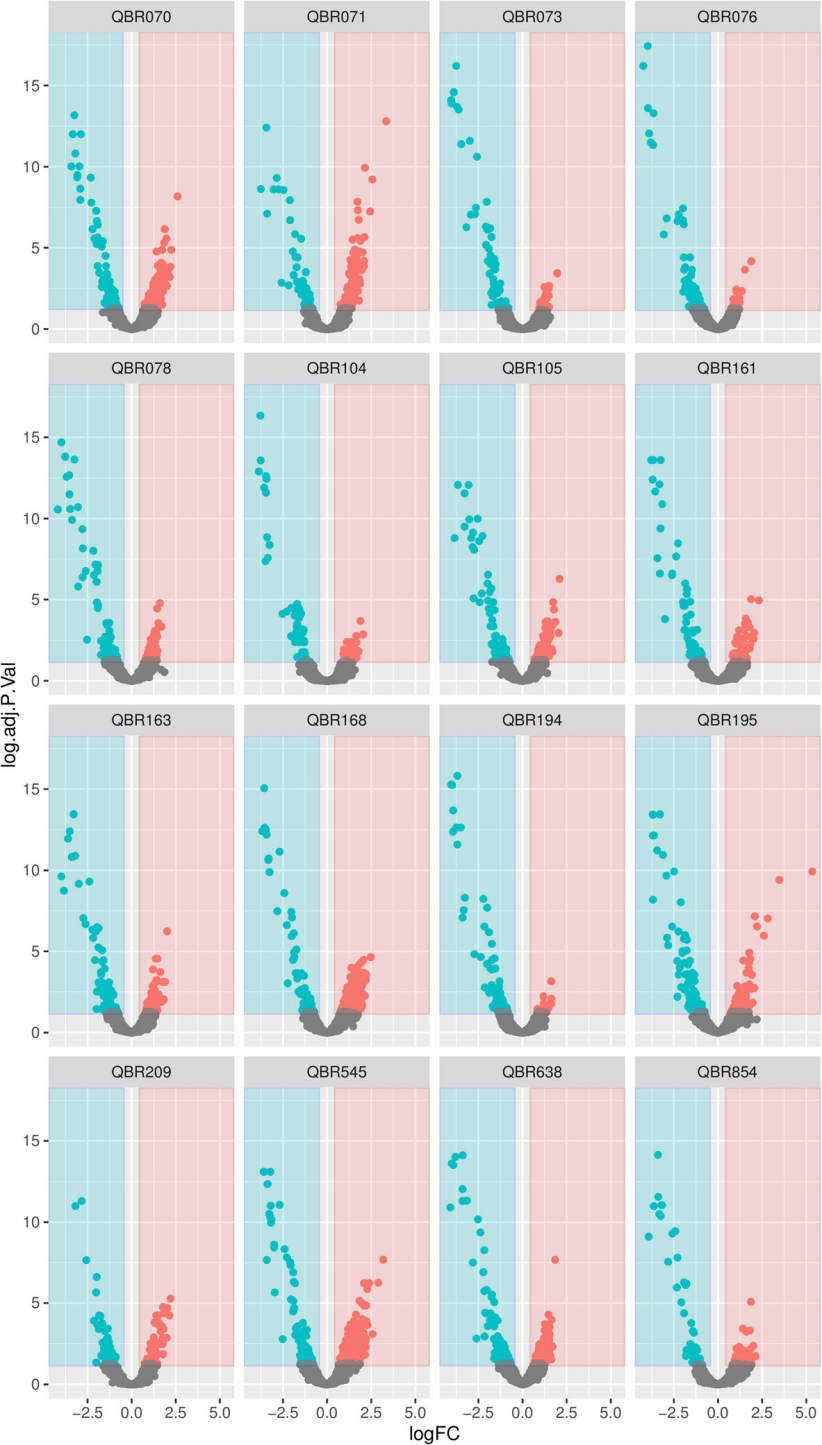
Volcano plots of genes showing altered expression in both natural isolates and mutants. Log2 fold changes and their corresponding *p*-values of all genes in the microarray were considered to build the Volcano plot. Genes up-regulated (>1.5 fold change, *p* < 0.05) are depicted in *red box*. Genes down regulated (>1.5 fold change, *p* < 0.05) appear in *blue boxes*. Genes that did not show a significantly modified pattern are represented in *grey dots*.

**Fig. 4:**
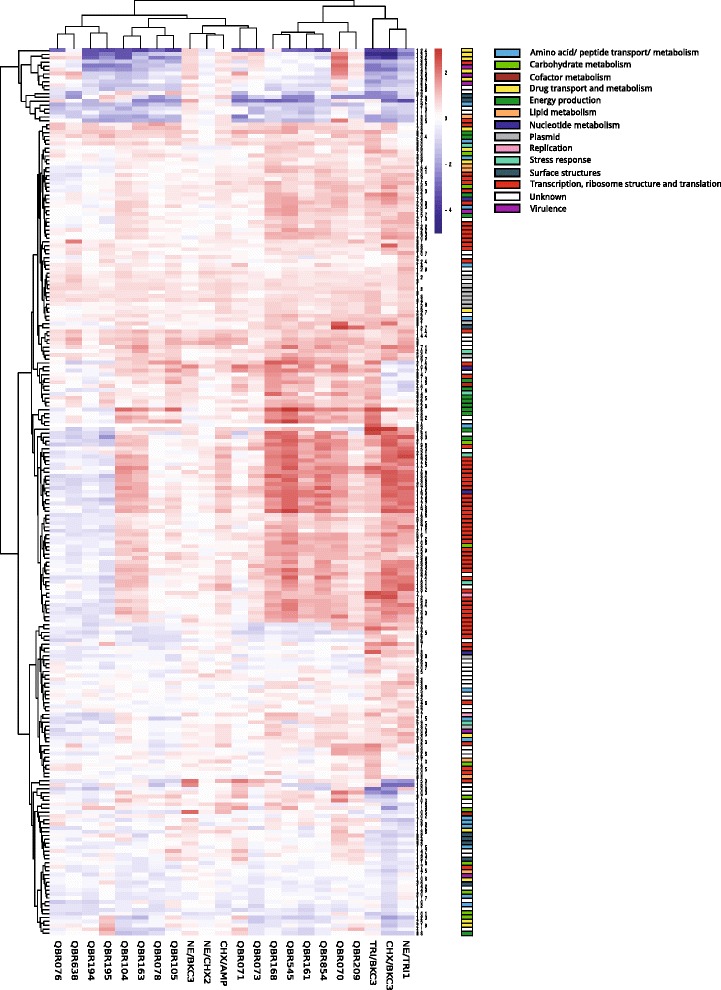
Heat map of the common genes with altered expression of natural isolates and mutants

The genes with altered expression in mutants were investigated for their expression in field isolates with decreased susceptibility to biocides (Fig. 3, Fig. 4 and Additional file 3: Table S2). Over and down expression of particular genes was noticed for both natural isolates and mutants (3 and -4 fold changes, respectively).

Similarly to what was observed for mutants, transcriptomic changes did not fully correlate with the phenotypes of susceptibility to biocides. However, enhanced expression of some of the same ribosomal proteins was identified for field strains. A higher number of metabolism-related genes were over-expressed in the wild-type isolates. Furthermore, the over-expression of genes encoding pathogenicity**-**related proteins (e.g. macrophage stimulating factor, flagellar hook-associated, factor PagK and membrane-related such as PgtE protease) occurred in different strains.

Other changes were only detected among wild-type strains. For example, the strain with the highest MBC for TRI (>512 mg/L) showed a high level expression of the ABC transporter *ydeY*, a pump for which a role on biocide resistance has not been described so far. Up-regulation of genes involved in the modification of the lipid A implicated in polymyxin resistance was found for some TRI^R^ and BKC^R^ strains. On the other hand, slight down-regulation of the *acrE* efflux pump was observed in three PFGE-unrelated strains exhibiting a susceptible biocide phenotype (TRI^S^/CHX^S^/BKC^S^) while one of them had the *sugE* gene over-expressed. A number of antibiotic resistance genes were up- or down-regulated in natural isolates.

### Resistance to biocides reduces the fitness of *Salmonella* SL1344

Variable values of fitness cost (FC) were observed for some mutants with disparate biocide phenotype (Table 1 and Additional file 1: Figure S2). Both NE/CHX2 (TRI^R^/BKC^R^/CHX^R^), which did not show any considerable susceptibility change, and TRI/BKC3 (TRI^HS^/BKC^R^/CHX^R^), showed the highest FC values (34 % and 31 %, respectively). They had deleted *bigA* gene. Mutants TRI^R^/BKC^R^/CHX^R^ (NE/TRI1), and TRI^HS^ (CHX/AMP) showed similar FC values (11 % and 16 %, respectively) (Additional file 1: Figure S2). Mutants fitter than parental strain showed increased MIC_CAZ_.

Besides changes in growth rates, altered colony morphology was also observed. Although several mutants had smoother and smaller colonies than the parental strain, we did not observe notable differences in the rdar (red, dry and rough) morphotype, an aggregative and resistant physiological state which has been linked to survival in nutrient-limited environments [38], or in the genomic *XbaI*-genomic digested DNA profiles (Additional file 1: Figure S3).

## Discussion

This paper documents a versatile adaptive response of the *Salmonella enterica* strain SL1344 after exposure to inhibitory concentrations of biocides or antibiotics which resulted on a diversity of phenotypes and genomic and proteomic changes. The emergence of mutants with phenotypes of antimicrobial resistance (antibiotics and/or biocides) which had not been previously exposed to antimicrobial agents but were recovered on selection plates, suggests that a variety of mutants with altered susceptibility to biocides easily arises. Biocides, acting on multiple cellular targets, would drive random selection of mutants, eventually causing pleiotropic changes; and therefore a high diversity of phenotypes were associated with biocide-tolerance. The fact that SHC did not select for mutants might provide a chance for efficient and safe sanitization.

Most mutants showed slight variations in their MIC values to the antimicrobials tested. Although cross-resistance between biocides and antibiotics is frequently described for biocide resistant mutants, we also observed increased susceptibility for some antimicrobials, a phenomenum that can be attributed to frequent collateral effects in the emergence of resistance as previously documented for antibiotic resistant bacteria [39, 40]. Indeed, antimicrobial resistant mutants of *Salmonella* presented increased susceptibility to envelope active inhibitory compounds. Importantly, this sort of compounds turns the cytoplasmic membrane more permeable, which often results on reduced viability. Therefore the ability to become resistant to these antimicrobials is lower. A part from the membrane vulnerability, most mutants potentially had alterations in the oxidative metabolism and protein synthesis. Our data showed that TRI resistance was often accompanied by higher tolerance to compounds of different antibiotic families, with the exception of aminoglycosides. This finding is in line with other studies in *Salmonella enterica* strains that reported increases in the susceptibility to aminoglycosides and CHX accompanying TRI^R^ [41, 42]. While most studies on the field focused on the analysis of cross-resistance of biocides and antibiotics, negative epistasis phenomena inferred from the simultaneous emergence of susceptibility to aminoglycosides or biocides and resistance to other antimicrobial agents are not uncommon [20, 39]. This antagonistic pleiotropy epistatic effect known as “collateral sensitivity” must be taken into consideration when evaluating the risks for the acquisition of resistance or to envisage methods for reducing or even eliminating resistant microorganisms in the field [40, 43].

Comparative genomic analysis revealed changes in a variety of genes, some of them previously linked to tolerance to antibiotics or metals (*fabI*, *yea* and *fts*) [44] and others newly identified here. Different amino acid changes at position 93 of the FabI protein resulted in TRI^R^ phenotypes [24, 32, 41, this study]. However, differences in the polarity of amino acids at position 93 might be associated with distinct structural conformations of FabI protein that would affect MIC_TRI_ values; higher values (MIC_TRI_ ≥ 2 mg/L for the mutant of this study) occurring when an uncharged amino acid (Gly or Val) is substituted by a polar amino acid (Ser).

Non-functional *ftsK* gene mutants have previously shown increased susceptibility to β-lactams and CIP and tolerance to chromate in *Pseudomonas aeruginosa* [45]. In this study mutants with SNPs in *ftsK* (NE/TRI1 and CHX/BKC3) showed either increased susceptibility to several β-lactams or to most CIP-related antibiotics, respectively. The *tolA* gene showed a mutation and differential expression in different mutants. Other SNPs may also modulate virulence in the mutants as for instance, mutants in *motB* (and *motA*) genes may paralyse the flagellar phenotype influencing adhesion and invasion of cells [46]. Flagellar assembly and/or mobility may antagonize the T3SS that delivers effectors into the host cell of some pathogens, revealing the potential impact of cross talk between some virulence factors depending on the bacterial colonization phase and infection type [47].

While particular changes at genetic level in the mutants were detected, a remarkable alteration of the expression profiles was noted in both mutants and field isolates, with overexpression of ribosomal protein synthesis as well the down-regulation of genes involved in global stress and regulatory mechanisms, metabolism of amino acids (lysine, asparagine, threonine), secondary metabolism, transport, virulence, chemotaxis, invasion pathways and of unknown function. This might indicate a higher cellular activity with lower virulence. Efflux pumps previously involved in biocide tolerance were up-regulated in some mutants and field isolates. They included SugE, classically implicated in QACs resistance and frequently found in *Salmonella* isolates of clinical and animal origin [48, 49] or AcrAB [41, 50], whose over-expression is known to contribute to antimicrobial resistance yet at low-level.

This is the first study characterizing both the genomic and expression profiles after antimicrobials challenge, although the stress response after exposure to a high diversity of environmental stressors including biocides or antibiotics has been tackled before. Cold-shock response, allowing the survival of *Listeria monocutogenes* in the presence of biocides was previously reported [10]. Similarly to the mutants selected in BKC in this study, antibiotic-resistant mutants obtained under different metabolic conditions were related to attenuated virulence due to low expression of the T3SS [51–53], or mutation in transcriptional regulators. The RNA polymerase regulates the transcription of genes encoding transport proteins and enzymes involved in the biosynthesis of the metabolic intermediates of exopolysaccharides, lipids, lipopolysaccharides, lipoproteins, flagella and peptidoglycan. This protein is stress-induced and plays a central role in the control of processes that involve physical interaction of an organism with the environment, as colonization of host surfaces (virulence) or biofilm formation [54]. Polymorphisms in genes coding for RNA polymerase subunit α (*rpoA,* described in mutants selected in QACs) [24, 50], and also σ factors (*rpoS* and *rpoD* genes, related to high-level resistance towards TRI) [55]) were previously reported. Despite other genes coding for RNA polymerase activity (*rpoZ* and *rpoB*) were shown here to be up-regulated, it corroborates the importance of this protein as a target implicated in intrinsic resistance to biocides [23, 55]. Conversely, down-regulation of transcriptional and ribosomal genes were previously detected after exposure to CHX [50]. In addition, we report less transcripts of members of the LuxR/UhpA and AraC family, the last one being negative regulator of HilA, the key regulator of the SPI1 [35, 36]. Down-regulation of genes encoding virulence-related proteins for several mutants might suggest a lower pathogenicity.

The finding of similar transcriptomic changes found in both biocide resistant mutants and field isolates with reduced susceptibility to biocides, suggests the involvement of common general responses that include diverse alterations in metabolic and chemotaxis pathways, protein synthesis, cell envelope or regulation of pathogenicity-islands, which has been reported in other studies analysing biocide-induced mutants of *Salmonella* and other species [9, 19, 56].

## Conclusions

In summary, this study shows that growth of *Salmonella* in the presence of selective concentrations of biocides or antibiotics leads to the selection of mutants with variable susceptibility to antimicrobials (“cross-resistance” or “collateral sensitivity”-like phenotypes) which is consistent with the “multiples target sites” hypothesis of most biocidal agents [57, 58]. The results highlight the wide range of pathways employed by *Salmonella* to counteract biocides and achieve stasis/stress survival. Unlike to what has been commonly reported, overexpression of AcrAB-like pumps did not seem to be the main mechanism involved in biocide tolerance. Detection of SNPs was not associated with altered expression of related genes, making data from genomic and transcriptomic analysis necessary for a comprehensive analysis of biocide-challenged strains. Finally, most selected biocide-resistant mutants presented fitness costs, an issue that might reduce their chances to spread under non-selective conditions.

## Additional files

Additional file 1: Figure S1. MIC distributions to triclosan, chlorhexidine and benzalkonium chloride for 62 natural *Salmonella* isolates. The number of *Salmonella* isolates with reduced susceptibility to biocides analysed for gene expression are indicated above the arrows and MIC susceptibility values. Colors are according biocide distributions. (*) an isolate showed simultaneously reduced susceptibility to CHX and BKC. Other 6 isolates more susceptible for biocides were analysed for control (TRI^S^/CHX^S^/BKC^S^: 0.06-0.12/2-8/32-64 mg/L).
**Figure S2.** *XbaI* digested-chromosomal DNA PFGE of several *Salmonella mutants* and its parental strain (5-35 s for 21 h).
**Figure S3.** Growth curves of *Salmonella* mutants and the parental strain in plain LB at 37 °C with shaking. (DOCX 446 kb) Additional file 2: Table S1. Differential gene expression of common genes from LIMMA and SAM methods for mutants. (XLSX 48 kb) Additional file 3: Table S2. Differential gene expression of common genes from LIMMA and SAM methods for natural isolates. (XLSX 280 kb)
