# Supplementary material for: Multiple adaptive routes of Salmonella enterica Typhimurium to biocide and antibiotic exposure
Source: BMC Genomics. 2016 Jul 13;17:491. doi: 10.1186/s12864-016-2778-z (PMC4943003; doi:10.1186/s12864-016-2778-z)

Figure S1. MIC distributions to triclosan, clorhexidine and benzalkonium chloride for the collection of 62 natural *Salmonella* isolates.


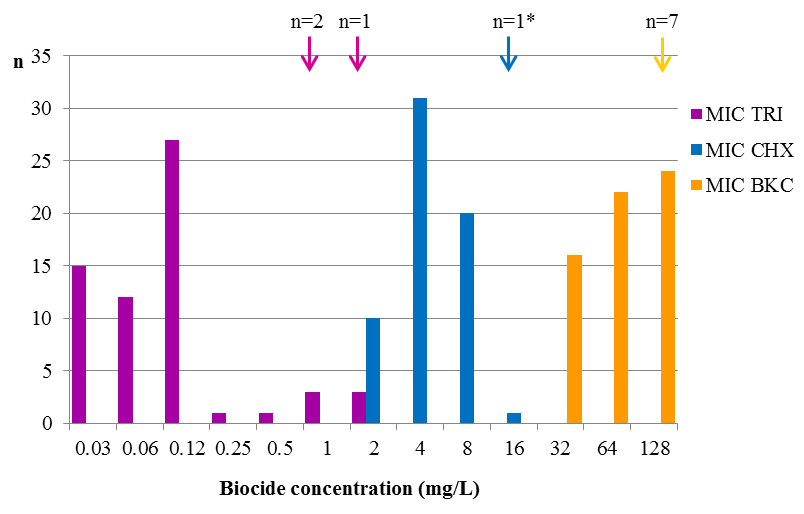


The number of *Salmonella* isolates with reduced susceptibility to biocides analysed for gene expression are indicated above the arrows and MIC susceptibility values. Colors are according biocide distributions. * corresponded to an isolate that showed simultaneously reduced susceptibility to CHX and BKC. For control other 6 isolates more susceptible for biocides were analysed (TRI^S^/CHX^S^/BKC^S^: 0.06-0.12/2-8/32-64 mg/L)

Figure S2. Growth curves of *Salmonella* mutants and the parental strain SL1344 in plain LB at 37ºC with shaking and respective growth parameters.


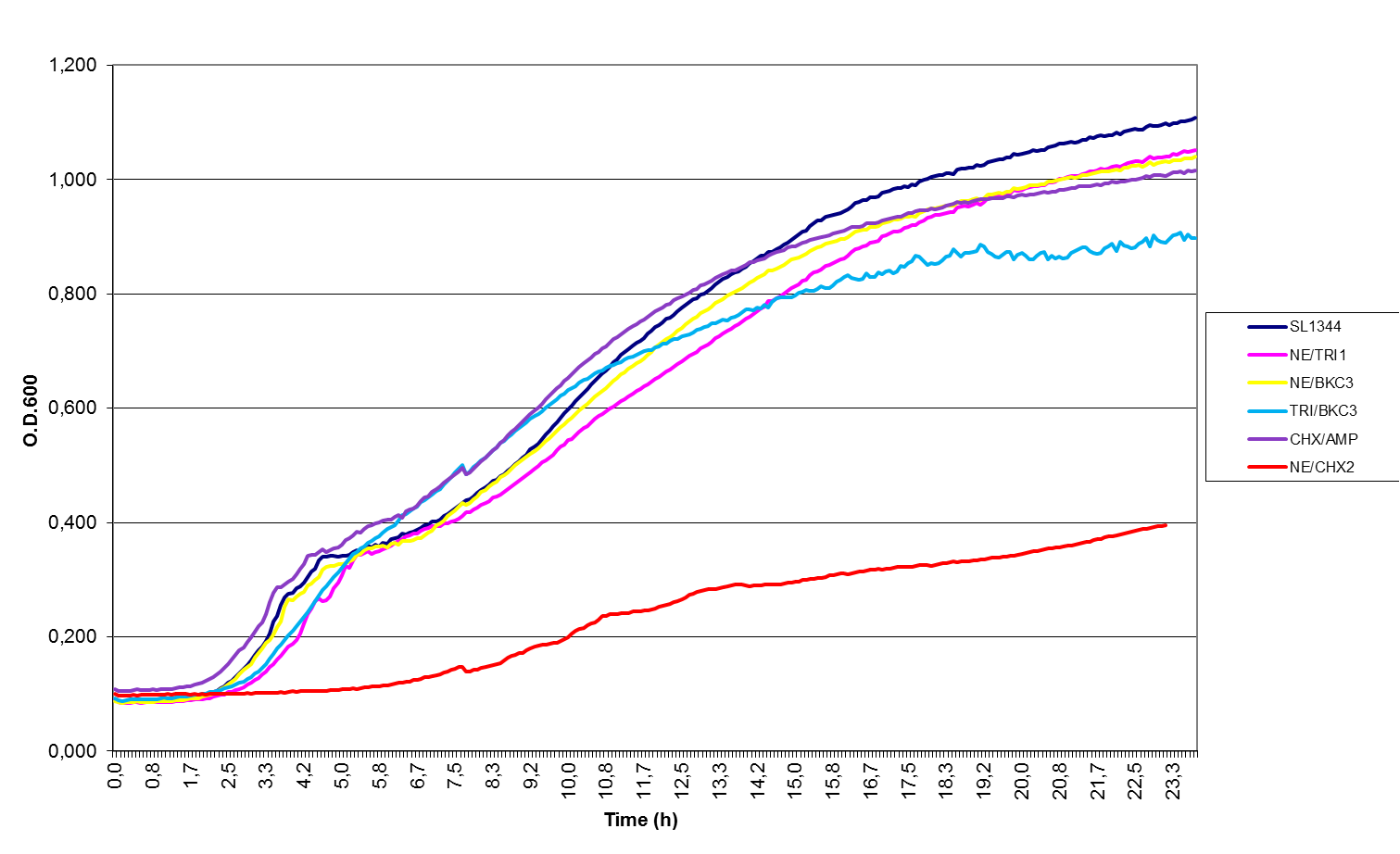


| **Strain** | **Growth Rate (GR)** | **R** | **Mean Relative GR** ± **S.D.** | **Fitness cost (%)** |
| --- | --- | --- | --- | --- |
| SL1344 | 0.014 | 0.996 |  |  |
| NE/TRI1 | 0.012 | 0.997 | 0.887 ± 0.063 | 11.3 |
| NE/BKC3 | 0.014 | 0.999 | 1.008 ± 0.075 | -0.8 |
| TRI/BKC3 | 0.009 | 1.000 | 0.688 ± 0.158 | 31.2 |
| CHX/AMP | 0.011 | 0.999 | 0.834 ± 0.130 | 16.6 |
| CIP/TRI1 | 0.032 | 0.995 | 1.631 ± 0.253 | -63.1 |
| NE/CHX2 | 0.013 | 0.992 | 0.663 ± 0.125 | 33.7 |

Figure S3. *XbaI* digested-chromosomal DNA-PFGE of several *Salmonella* mutants and the parental strain (5-35s for 21h).


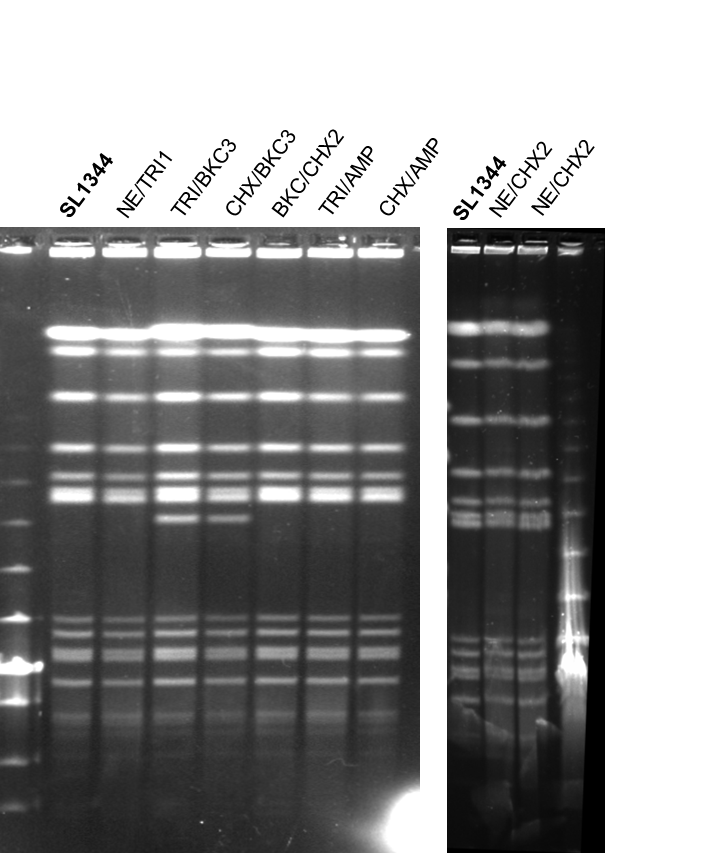

Supplement: Additional file 1: Figure S1. — MIC distributions to triclosan, chlorhexidine and benzalkonium chloride for 62 natural Salmonella isolates. The number of Salmonella isolates with reduced susceptibility to biocides analysed for gene expression are indicated above the arrows and MIC susceptibility values. Colors are according biocide distributions. (*) an isolate showed simultaneously reduced susceptibility to CHX and BKC. Other 6 isolates more susceptible for biocides were analysed for control (TRIS/CHXS/BKCS: 0.06-0.12/2-8/32-64 mg/L). Figure S2. XbaI digested-chromosomal DNA PFGE of several Salmonella mutants and its parental strain (5-35 s for 21 h). Figure S3. Growth curves of Salmonella mutants and the parental strain in plain LB at 37 °C with shaking. (DOCX 446 kb) [file 12864_2016_2778_MOESM1_ESM.docx]
